# SCNrank: spectral clustering for network-based ranking to reveal potential drug targets and its application in pancreatic ductal adenocarcinoma

**DOI:** 10.1186/s12920-020-0681-6

**Published:** 2020-04-03

**Authors:** Enze Liu, Zhuang Zhuang Zhang, Xiaolin Cheng, Xiaoqi Liu, Lijun Cheng

**Affiliations:** 1 Department of BioHealth Informatics, School of Informatics and Computing, Indiana University—Purdue University, Indianapolis, IN 46202 USA; 2 Department of Toxicology and Cancer Biology, College of Medicine, University of Kentucky, Lexington, KY 40536 USA; 3 College of Pharmacy, Division of Medicinal Chemistry and Pharmacognosy, the Ohio State University, Columbus, OH 43210 USA; 4 Department of Biomedical informatics, College of medicine, the Ohio State University, Columbus, OH 43210 USA

**Keywords:** Integrated network, Protein-protein interaction network, Spectral clustering, Drug target ranking

## Abstract

**Background:**

Pancreatic ductal adenocarcinoma (PDAC) is the most common pancreatic malignancy. Due to its wide heterogeneity, PDAC acts aggressively and responds poorly to most chemotherapies, causing an urgent need for the development of new therapeutic strategies. Cell lines have been used as the foundation for drug development and disease modeling. CRISPR-Cas9 plays a key role in every step-in drug discovery: from target identification and validation to preclinical cancer cell testing. Using cell-line models and CRISPR-Cas9 technology together make drug target prediction feasible. However, there is still a large gap between predicted results and actionable targets in real tumors. Biological network models provide great modus to mimic genetic interactions in real biological systems, which can benefit gene perturbation studies and potential target identification for treating PDAC. Nevertheless, building a network model that takes cell-line data and CRISPR-Cas9 data as input to accurately predict potential targets that will respond well on real tissue remains unsolved.

**Methods:**

We developed a novel algorithm ‘Spectral Clustering for Network-based target Ranking’ (SCNrank) that systematically integrates three types of data: expression profiles from tumor tissue, normal tissue and cell-line PDAC; protein-protein interaction network (PPI); and CRISPR-Cas9 data to prioritize potential drug targets for PDAC. The whole algorithm can be classified into three steps: 1. using STRING PPI network skeleton, SCNrank constructs tissue-specific networks with PDAC tumor and normal pancreas tissues from expression profiles; 2. With the same network skeleton, SCNrank constructs cell-line-specific networks using the cell-line PDAC expression profiles and CRISPR-Cas 9 data from pancreatic cancer cell-lines; 3. SCNrank applies a novel spectral clustering approach to reduce data dimension and generate gene clusters that carry common features from both networks. Finally, SCNrank applies a scoring scheme called ‘Target Influence score’ (TI), which estimates a given target’s influence towards the cluster it belongs to, for scoring and ranking each drug target.

**Results:**

We applied SCNrank to analyze 263 expression profiles, CRPSPR-Cas9 data from 22 different pancreatic cancer cell-lines and the STRING protein-protein interaction (PPI) network. With SCNrank, we successfully constructed an integrated tissue PDAC network and an integrated cell-line PDAC network, both of which contain 4414 selected genes that are overexpressed in tumor tissue samples. After clustering, 4414 genes are distributed into 198 clusters, which include 367 targets of FDA approved drugs. These drug targets are all scored and ranked by their TI scores, which we defined to measure their influence towards the network. We validated top-ranked targets in three aspects: Firstly, mapping them onto the existing clinical drug targets of PDAC to measure the concordance. Secondly, we performed enrichment analysis to these drug targets and the clusters there are within, to reveal functional associations between clusters and PDAC; Thirdly, we performed survival analysis for the top-ranked targets to connect targets with clinical outcomes. Survival analysis reveals that overexpression of three top-ranked genes, PGK1, HMMR and POLE2, significantly increases the risk of death in PDAC patients.

**Conclusion:**

SCNrank is an unbiased algorithm that systematically integrates multiple types of omics data to do potential drug target selection and ranking. SCNrank shows great capability in predicting drug targets for PDAC. Pancreatic cancer-associated gene candidates predicted by our SCNrank approach have the potential to guide genetics-based anti-pancreatic drug discovery.

## Introduction

Pancreatic cancer is the third leading cause of cancer death in the United States. The American Cancer Society estimates that 53,070 Americans will be diagnosed with pancreatic cancer in 2017, and that 41,780 will die from the disease [1]. About 85% of pancreatic cancers are pancreatic ductal adenocarcinomas (PDACs). Despite decades of effort, PDAC has the shortest survival time of all major cancers, and the five-year survival rate is only ~ 8%. Patients diagnosed with PDAC are usually diagnosed at advanced stages, when tumor cells have spread into the lymphatic system and vicinal organs, which limit the choices of effective treatments [2]. Another challenge in treating PDAC is its treatment-recalcitrant characteristics [3, 4], which often lead to insensitivity towards many chemotherapeutic drugs and target-based drugs [5]. Even though drug combinations such as Gemcitabine plus epidermal growth factor receptor (EGFR) inhibitor Erlotinib or Gemcitabine plus Nab-paclitaxel have been widely applied in the clinical setting, survival is only modestly improved [3]. Therefore, identifying novel drug targets for treating PDAC is an urgent need.

The establishment of cell lines from human tumors is largely responsible for our early progress in cancer research. Cancer cell models show immense potential for cancer medicine by linking cellular variation to genomic features. However, the complexity of modeling cancer in cells has increased the difficulty of observing and manipulating a complex PDAC process in a manner that cannot be performed in patients [6]. In recent years, CRISPR-Cas9 genome editing technology has become a reliable tool for discovering therapeutic targets in cancer cells and validating large-scale preclinical testing on cancer cells [7]. This ease of construction of CRISPR libraries enables large-scale screening that targets all (or a desired subset) of the protein-coding genes encoded in a whole genome by microarray-based platform [8]. The capabilities of CRISPR-based genetic screens offers great opportunities to observe cell variations, which further benefit essential gene selection and effective target identification on cancer cells. On the other hand, recent advances in high-throughput microarrays have produced a wealth of information concerning pancreatic cancer mechanisms. Whole genome profiling has allowed the simultaneous identification of hundreds of genes that are perturbed in pancreatic cancer patients. Substantial progress has been made in our understanding of the biology of pancreatic cancer from the molecular level, including cancer-associated genes for drug targets in PDAC [9]. However, it remains a challenge to identify potential targets by building upon cancer cell CRISPR/Cas9 genetic perturbation screen data and transcriptome data collected from patients and cancer cells.

Network-based analysis has greatly benefited cancer biology. Patterns that reflect important cancer-related processes and mechanisms can be shown in a large-scale complex network, in which genes, proteins and other components interact with each other. A better understanding of associations/regulations of genes or proteins from a network perspective can provide valuable insights towards target selection for developing novel cancer treatments [10]. So far, biological networks have been widely used in numerous studies for identifying genes related to certain therapies through a curated database, specialized drug-protein [11] or protein-disease networks [12, 13]. (1) Curated databases, such as STRING protein-protein interaction [14] network and KEGG [15] pathway network, provide complete genome-wide networks that contain entire gene regulations, signal transductions and gene-protein associations. However, these methods are not built for specific cancer types, making them too generalized. It is also difficult for people to analyze them as a whole. (2) A drug-protein network is often used to investigate the mechanism of drug action and drug target prioritization [16]. For instance, Isik et.al provided drug target identification by perturbed gene expression from Connectivity Map (CMAP) [17] and protein-protein Interaction (PPI) network information. However, these technologies did not directly connect a drug with disease genes. (3) Constructing protein-disease networks is another approach to identify gene-disease associations for selecting therapeutic targets in cancer [18]. Ferrero et al. proposed a semi-supervised network approach, which evaluates disease association evidence and makes de novo predictions of potential therapeutic targets based on that [19]. These types of methods fail to incorporate target information in their models to accurately predict drug targets.

CRISPR-Cas9 genome-wide perturbation data provides the opportunity to find genes vital to pancreatic cancer by looking at the mortality of an individual gene. The mortality of an individual gene is found from observing genes expression variation of cancer cells [20]. However, solely using gene perturbation data targets cannot resolve target ranking problems. Moreover, identifying drug targets that actually work on living tissues from gene perturbation data is still challenging. In this paper, we proposed a method called ‘SCNrank’ that systematically utilizes expression data from tissue and cell-line, along with gene perturbation data and PPI networks to select and rank druggable targets that effectively work on tissues. SCNrank systematically compares the network structure between PDAC tissue-specific network and PDAC cell-line-specific network to identify similarities commonly exited in two networks. PDAC then utilized CRISPR-Cas9 data to score and rank targets from these similarities. To our knowledge, this is the first-time people have proposed a model that systematically score and rank potential targets by considering network similarities between tumor networks and cell-line networks. On the other hand, we validated ranking drug targets by 1) mapping them onto existing PDAC drug targets; 2) applying pathway analysis on drug targets and the clusters within to show their functional associations with PDAC; and 3) performing survival analysis for top ranked drug targets.

## Materials and methods

### Research framework

**Fig. 1:**
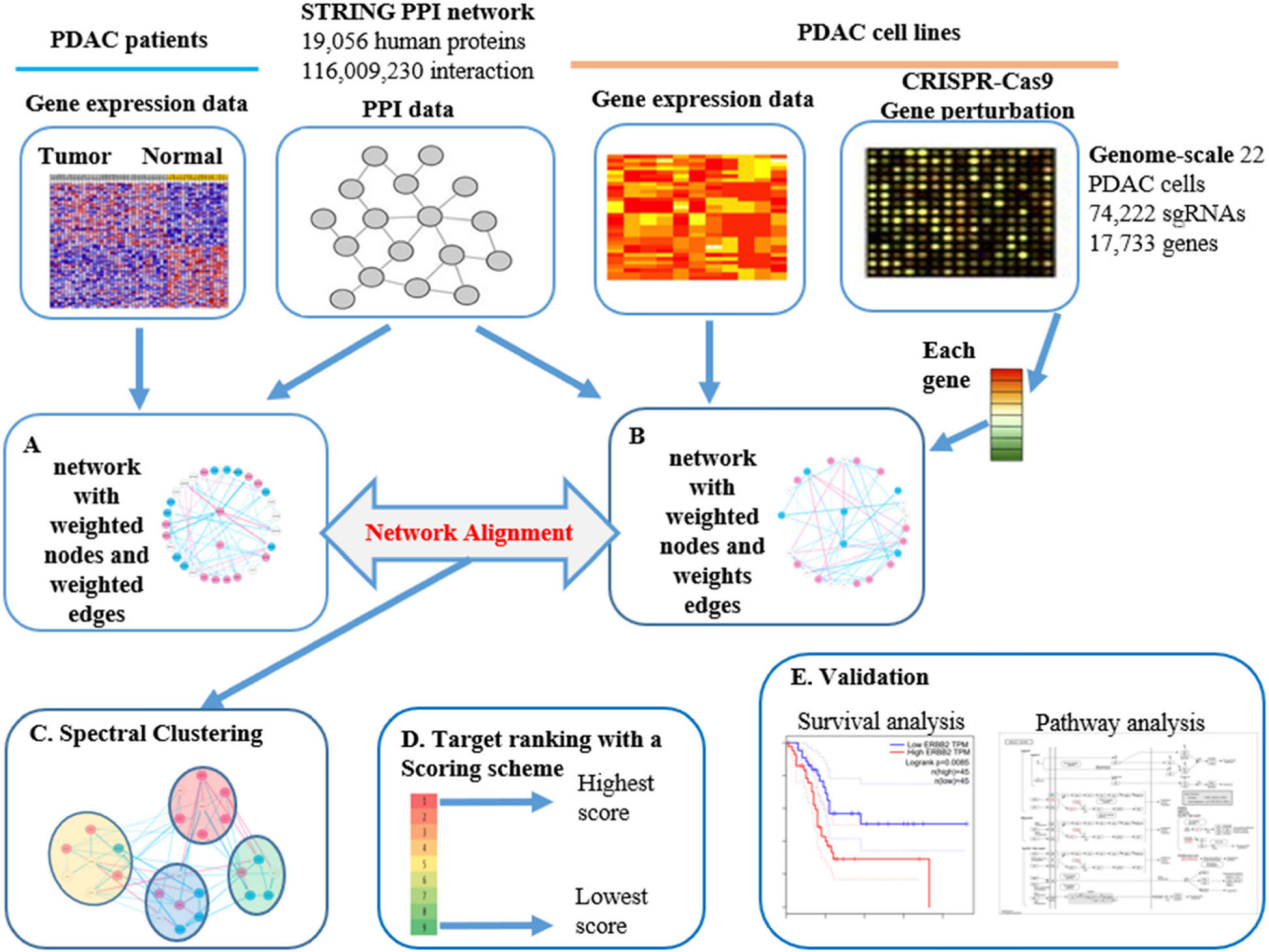
Workflow of this study (**a**) Constructing an integrated tissue-specific PDAC network with weighted nodes and weighted edges using tissue PDAC expression profile, normal PDAC expression profile and PPI network data. **b** Constructing an integrated cell-line-specific PDAC network with weighted nodes and weighted edges using cell-line PDAC expression profile, CRISPR data and PPI network data. **c** Spectral clustering for integrated tissue-specific PDAC network. **d** Aligning clustering results on integrated cell-line-specific PDAC network and ranking targets with a scoring scheme (TI score). **e** Validation on top ranked targets.

This study aimed to identify perturbed genes based on gene expression datasets representing distinct states of tumor tissues and adjacent normal tissues, and then align them with the integrated network that is generated from cell-line PDAC expression and CRISPR-Cas9 perturbation data for target selection (Fig. 1). Gene expression profiles from 263 samples, CRISPR-Cas9 data from 22 pancreatic cancer cell-lines, STRING protein-protein interaction (PPI) network consisting of 19,056 proteins and 116,009,230 PPI, and 1317 targets corresponding to all FDA-approved drugs are included in this study. We developed a subnetwork target identification algorithm called Spectral Clustering for Network-based target Ranking ‘SCNrank’. The core idea of SCNrank is to align tissue PDAC patterns to cell-line PDAC, and then incorporate gene perturbation (CRISPR-Cas9 data) to score and rank targets based on these patterns.

**Fig. 2:**
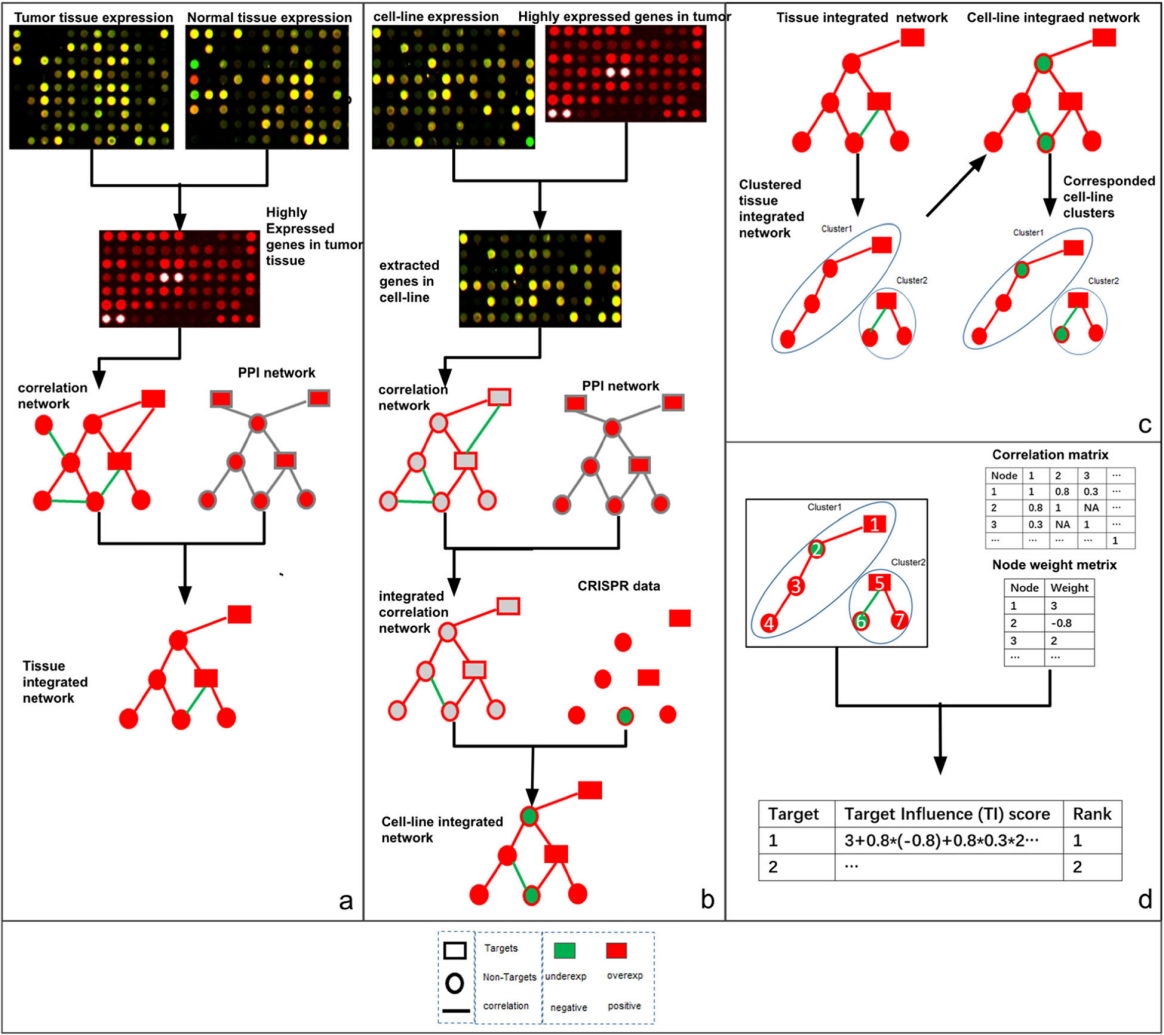
Workflow of ‘SCNrank’ (**a**) Constructing integrated tissue PDAC network; (**b**) Constructing integrated cell-line PDAC network; (**c**) Spectral clustering for subnetwork partitioning; (**d**) Clusters alignment between tissue network and cell-line network, and then calculating TI score for targets to rank them

SCNrank used STRING PPI [14] as a skeleton and expression data from PDAC tissue and cell-line as complements to construct two networks: one for tissue and one for cell-line, both of which share the same PPI skeleton but have totally different weights of nodes and edges, which are used to carry their unique characteristics. We took advantage of dimension reduction approaches to decompose the networks into clusters to better capture their common features in tissue network to detect optimal targets. Finally, we aligned the clusters of interest from tissue network to cell-line networks and then applied a customized Dijkstra-paths searching algorithm for searching and ranking all possible targets with each cluster. SCNrank includes four steps (see Fig. 1a-d): For cell-line PDAC and Tumor PDAC data respectively, the algorithm generates integrated networks and maps them onto the STRING PPI network so that they become comparable (Fig. 1a-b). Subnetwork partition (Fig. 1c) and a scoring scheme for aligned subnetworks (Fig. 1d) are two key methods of SCNrank. Validations on ranked targets are included in this study (Fig. 1e). The detailed SCNrank algorithm is illustrated in Fig. 2.

Subnetwork partitioning is typically used to subdivide large networks into smaller, more efficient subnetworks. Subnetworks can reflect important cancer-related gene regulation processes and module mechanisms. Associations/regulations of genes or proteins from subnetworks can provide valuable insights towards target selection for developing novel treatments for pancreatic cancer [10]. Spectral clustering [21] is a dimension reduction and clustering graphs approach. It firstly reduces data dimension so that core features will be revealed, then performs clustering analysis on the simplified data to better categorize data compared to approaches that directly perform clustering on the complete data. In our study, spectral clustering is used to: firstly reduce data dimension for the integrated networks; then perform clustering for the integrated networks. Here, spectral clustering is designed to reduce data dimension and identify perturbed gene subnetworks of pancreatic tumors, where nodes are originated from dysregulation degree of gene expression datasets representing distinct states tumors and adjacent tumors normal, edges are from correlation coefficient of tumors gene expression profiles.

#### Subnetwork alignment score for priority targets

Numerous graph alignment approaches by certain features or conditions have been developed. Typically, algorithms include seed-based and score-based strategies. SubNet [22] firstly apply seed genes with the PageRank algorithm to identify aligned subnetworks [23]. Score-based strategies rely on the scoring schemes on either edges or nodes. Guo et al. proposed a condition-specific subnetwork selection algorithm that scores solely edges [24]. Dezso et al. developed an algorithm that scores nodes to extract disease-specific subnetworks [25]. However, this method only uses parts of graph information, such as nodes or edges to detect network structure variation, which is not enough to observe network topology variation [26]. IODNE deploys a minimum spanning tree search algorithm and simultaneously scores edges and nodes for selecting subnetworks that are most dysregulated for potential target disease genes. IODNE can then be successfully applied for breast cancer subnetwork identification [27]. However, it doesn’t provide direct evidence of actionable drug targets. To overcome these drawbacks, we developed a scoring scheme that simultaneously takes node weight and edge weight into account. *Dijkstra* shortest paths algorithm is proposed to rank subnetworks for ranking targets.

### SCNrank algorithm

‘SCNrank’ takes multiple types of omics data from tissue and cell-line data as input to rank druggable targets. SCNrank mainly consists of four steps (shown as subgraphs A, B, C, D in Fig. 2).

#### STEP A: construct an integrated network for tissue PDAC

The algorithm first compares tumor tissue and normal tissue expression profiles to select the overexpressed genes in tumors. Since the sample number of tissue tumor and normal groups are not equal, we performed an unpaired T-test with a *p*-value cut-off 0.05. Log fold changes between tumor and normal tissue samples are calculated for all significantly overexpressed genes. The algorithm then constructed a correlation network by calculating the Pearson correlation coefficient as edge weights. Log fold change is then used as the node weights in the network. The algorithm then maps the integrated network onto the STRING PPI network and selects the overlapped subnetwork. The rationale of mapping is that: 1. we believe high correlations among genes that also reflect on protein level are more likely to be true; 2. mapping both tissue integrated network and cell-line tissue integrated network onto the same PPI network makes them comparable via the PPI network. Eventually, a network with the skeleton from PPI network, edge weights from pair-wise gene correlation, and node weights from Tumor -versus-Normal log fold change are constructed.

#### STEP B: construct an integrated perturbation network of pancreatic cancer cells

Only genes that are selected in STEP 1 are picked from the cell-line expression profile for integrated network construction. Similarly, the pair-wise Pearson correlation coefficients for these genes are calculated to build a correlation network. The network is then mapped onto the STRING PPI network and only the overlapped subnetwork is kept. Gene essentiality value (CRISPR-Cas9 data) is then integrated into the network as node (gene) weights. Finally, two constructed networks share the same nodes and edges but with totally different node weights and edge weights.

#### STEP C: dimension reduction and network partition

Spectral clustering [21] is a dimension reduction scheme that divides a network into pieces based on the spectrum (eigenvalues) of the corresponding similarity matrix. In the clustering process, the high dimension network is reduced to low dimension clusters since common features among variables can be better captured from a graph perspective. Given a graph *G* with *n* nodes and *k* categories, the objective function of spectral clustering can be described as:

1 $$ \mathit{\min}. cut\left({A}_1,\dots {A}_k\right)=\frac{1}{2}\sum \limits_{i=1}^kW\left({A}_{i,}\overline{A_i}\right) $$

Where $W\left({A}_{i,}\overline{A_i}\right)$ is the weight between cluster *A*_*i*_ and its complement set $\overline{A_i}$. However, this has been proven as an NP-hard discrete problem. In this study, we applied a widely used spectral approach called RatioCut [28] to make the optimal cut by solving the following objective function:

2 $$ \underset{A_1,\dots, {A}_k}{\min}\mathrm{Tr}\left({A}^{\prime } LA\right)\ \mathrm{subject}\ \mathrm{to}\ {A}^{\prime }A=I $$

Where *L* is the normalized Laplacian matrix (defined as formula (7)), $A=Y{\left({Y}^TY\right)}^{-\frac{1}{2}}$ is a scaled partition matrix, and *Y* is a partition matrix indicating a clustering scheme.

The general steps of performing spectral clustering can be described as:

1. For *n* variables (nodes), construct an affinity matrix *S*

3 $$ S=\left(\begin{array}{ccc}{s}_{11}& \cdots & {s}_{1n}\\ {}\vdots & \ddots & \vdots \\ {}{s}_{n1}& \cdots & {s}_{nn}\end{array}\right) $$

Where *S*_*ab*_ in the matrix indicates the connectivity between variables *a* and *b* in the network.

2. Construct a diagonal matrix *D* as a degree matrix

4 $$ D=\left(\begin{array}{ccc}{d}_1& \cdots & 0\\ {}\vdots & \ddots & \vdots \\ {}0& \cdots & {d}_n\end{array}\right) $$

Where *d*_*a*_ in the matrix indicates the degree (total edges) of variable a in the network. Clearly,

5 $$ {d}_a=\sum \limits_{k=1}^n{S}_{ak} $$

3. Construct Laplacian matrix

6 $$ L\hbox{'}=D-S $$

4. Normalize the Laplacian matrix

7 $$ L={D}^{-\frac{1}{2}}{L}^{\prime }{D}^{-\frac{1}{2}} $$

5. Perform singular value decomposition for matrix *L*
6. Pick top *K* eigenvalues and their corresponding eigenvectors to generate a *N* ∗ *K* matrix
7. Perform *K*-means clustering [29] on the extracted matrix.

Clearly, the Laplacian matrix *L* consists of two types of node information: local information, which is node connectivity towards its neighbors in matrix *S*, and global information, which is node degrees, or ‘influence’ towards the entire network. Hence, the clustering strategy can be thought of as selecting similar nodes based on their local and global similarities. Inspired by this idea, we used the Pearson Correlation Coefficient (CC) among nodes instead of the connectivity value (0 or 1) in the affinity matrix to measure the local similarities among genes. We also plugged in log fold change of tumor versus normal expression value in the degree matrix to indicate the global influence of genes. Hence, matrix S and D becomes:

8 $$ {S}^{\prime }=\left(\begin{array}{ccc}{r}_{11}& \cdots & {r}_{1n}\\ {}\vdots & \ddots & \vdots \\ {}{r}_{n1}& \cdots & {r}_{nn}\end{array}\right) $$

and

9 $$ {D}^{\prime }=\left(\begin{array}{ccc}{FC}_1& \cdots & 0\\ {}\vdots & \ddots & \vdots \\ {}0& \cdots & {FC}_n\end{array}\right) $$

Where *r*_*ab*_ is the CC between gene a and b in the expression profile when constructing the integrated tumor network and integrated cell-line network.

*FC*_*a*_ is the log fold change of the gene when comparing its expression value in the tumor group to its value in the normal group while constructing the integrated tumor network. In the cell-line network, *FC*_*a*_ represents the gene essentiality value (CRISPR-Cas 9 value).

To fulfill formula (5), S needs to be normalized to:

10 $$ {S}^{\prime \prime }=\left(\begin{array}{ccc}\frac{r_{11}{FC}_1}{\sum_{k=1}^n{r}_{1k}}& \cdots & \frac{r_{1n}{FC}_1}{\sum_{k=1}^n{r}_{1k}}\\ {}\vdots & \ddots & \vdots \\ {}\frac{r_{n1}{FC}_n}{\sum_{k=1}^n{r}_{nk}}& \cdots & \frac{r_{nn}{FC}_n}{\sum_{k=1}^n{r}_{nk}}\end{array}\right) $$

Hence, the final Laplacian becomes *L*^′^ = *D*^′^ − *S*^′′^, and normalized Laplacian becomes

11 $$ L={D}^{\prime -\frac{1}{2}}{L}^{\prime }{D}^{\prime -\frac{1}{2}} $$

For K-means clustering, picking the optimal K could be arbitrary. In our case, K is equal to the number of eigenvalues that the algorithm picked. Too many or too few eigenvalues will result in overfitting and underfitting, respectively. Hence, we applied an intuitive approach: from K = 1 to the total number of variables, we performed a K-means algorithm and calculated Hartigan’s number, which is a measurement of the clustering quality, by comparing two clustering results. For a K-means clustering, if the number is greater than 10, then having K + 1-means clustering is of value [30]. We selected the K when the Hartigan’s number is firstly less than 10. We understand that this scheme of picking K doesn’t guarantee global optima.

#### STEP D: graph structure similarity alignment between subnetworks of dysregulation genes in tumors and perturbation networks in cancer cells and score to rank for priority potential targets

We applied spectral clustering on the tissue integrated network to look for genes that show common features. We then mapped 1317 targets (genes) for all FDA approved drugs onto clusters. Then, for the successfully mapped drug targets, we examined the influence that the target might have over whole clusters. We assumed that a drug target’s ‘influence’ is limited to its cluster. In that case, a drug target’s influence towards any node is determined by the paths between them. Hence, given a graph *G* (*V*, *E*), where *V* and *E* are node set and edge set, one can assume that the node weight set is *W* and edge weight set is *Y*. For a drug target *x*, its maximum ‘*influence’* towards all other nodes can be described as:

12 $$ \sum \limits_{k\in V}{W}_k\prod \limits_{i=k}^x{Y}_{i,{i}_{next}} $$

$$ where\ {Y}_{i,i\_ next}\in \mathrm{E} $$

Where $\prod \limits_{i=k}^x{Y}_{i,i\_ next}$ indicates the transmitted influence from target *x* to a node *k* via one possible path. Obviously, to maximize term (12), for every other node *i*, we need to find the most correlated path between *x* and *i*. Thus, the total influence of x becomes:

13 $$ \boldsymbol{TI}=\sum \limits_{k\in V}{W}_k\max \left(\prod \limits_{i=k}^x{Y}_{i,{i}_{next}}\right) $$

Here, the term $\max \left(\prod \limits_{i=k}^x{Y}_{i,{i}_{next}}\right)$ represents the most correlated path between x and i. And we define term (13) as Target Influence score (TI). We then developed a scoring scheme for calculating *TI* for all 367 drug targets. The detail is described in Table 1:

**Table 1:**
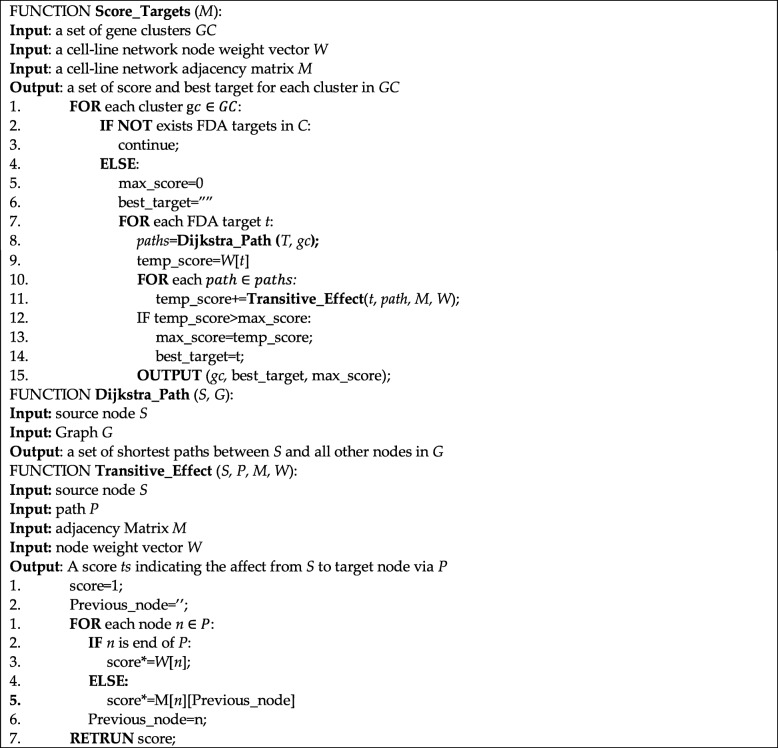
Scoring scheme for identifying druggable targets from a clustered graph

Given a source node and a graph, the famous *Dijkstra* algorithm [31] can find all shortest paths (a path that contains minimum weight) between the source node and all other nodes. By taking the reciprocal value for all edge weights, *Dijkstra* can be used to find the ‘heaviest’ paths, which is also the most correlated path, between the source node and all other member nodes in a cluster (a subnetwork). Thus, we applied the *Dijkstra* algorithm to find ‘most correlated’ paths between drug targets and all other genes within a cluster. The hypothesis behind it is that we believe when a drug target is aimed, the influence transmits to other nodes via most correlated paths. Moreover, in the cell-line specific network, two genes might be either positively or negatively correlated. Hence, we multiplied these correlations to allow the drug target to be positively or negatively associated with the other nodes. This multiplied coefficient will be multiplied by the node weight (gene essentiality value) to represent this node’s reaction towards the knockdown/knockout of the drug target as the node’s influence score. Finally, all influence scores within the cluster are summed up as the total influence score of the drug target for the entire cluster. Since most of the drug targets are highly regulated in tumors, we record the maximum score that a target can have towards its cluster. If multiple targets are in one cluster, we report the target that causes maximum influence on its cluster as the druggable target for that cluster. Finally, we ranked all targets by these influence scores. HMMR and POLE2 have been together reported as significantly overexpressed in PDAC and lung cancer from a large cohort.

### Materials

#### Expression data of PDAC

Expression data gathered from 263 samples across three groups are used in this study, including 92 PDAC cell-line samples, 113 PDAC tissue samples and 58 adjacent normal pancreas tissue samples. These data are all from the Gene Expression Omnibus (GEO, http://www.ncbi.nlm.nih.gov/geo/) database and are all generated from Affymetrix Human Genome U133 Plus 2.0 Array, which contains 54,675 probes pointing to over 20,000 genes. Complete annotation of all samples can be found in Additional file 1, (Table 2).

**Table 2:** Gene expression data used in this study along with their session Number in GEO database

Human pancreatic cancer cell line	Human PDAC tumors	Human normal pancreas tissues
GSE36133 (43)	GSE42952 (33)	GSE46385 (3)
GSE46385 (7)	GSE51978 (2)	GSE16515 (16)
GSE21654 (22)	GSE16515 (36)	GSE15471 (39)
GSE17891 (20)	GSE15471 (39)	
	GSE23952 (3)	
92 samples	113 samples	58 samples

#### Protein-protein interaction network

STRING [14] is a comprehensive and public pathway database (https://string-db.org/), which accumulates prior knowledge of biological pathways and protein-protein interactions. We included STRING network protein links version 11 data in our analysis.

#### Genome-wide CRISPR-Cas9 screening data and gene essentiality value

To measure gene essentiality, we used CRISPR-Cas9 v3.3.8 screening data from ‘Project Achilles’ [32–34], (https://portals.broadinstitute.org/achilles) which includes genome-wide CRISPR-Cas9 screening data that affect cell survival across 43 tumorous cell lines and genome-wide RNAi screening data over 501 cell-lines. We choose CRISPR-Cas9 over RNAi because recent studies have indicated that compared to RNAi, CRISPR-Cas9 has less off-target effects, and is thus better for cancer drug-target related research [35]. In total, gene perturbation data of 74,222 sgRNAs on 17,733 genes across 22 PDAC cell-lines are included in this study.

#### FDA approved drug targets

We downloaded all FDA approved drugs and their targets from Drug bank [5]. In total, all targets have been mapped onto 1317 genes, of which 283 genes are cancer drug targets.

### Data preprocessing

#### Gene expression profiles preprocess

We converted raw data (.cel files) to expression value by 3 steps: background correction, normalization and summarization. We then applied normalizations for all samples to make them comparable. Probe-based expression value is converted to gene-based expression value by sequentially applying the following settings: 1. A probe containing more than 20% missing data is eliminated; 2. A K-nearest neighbor approach (KNN) is applied to infer the missing data; We estimated the missing data with the average value from K = 10 nearest neighbors. 3. We convert probes to genes using Affymetrix U133 Plus 2.0 annotation file as a reference, which can be downloaded from AFFYMETRIX official website. For probes that point to the same gene, their value is averaged to represent the expression value of this gene.

#### Expression data normalization

To make the expression profiles from different samples comparable, We applied Microarray Suite 5 method (MAS 5.0) normalization algorithm [36], which is embedded in R package ‘affy’ available in Bioconductor (http://bioconductor.org). We then applied a Quantile Normalization for all expression samples to reduce the batch effect. Finally, all values are Log2 transformed and ready for analysis.

#### CRISPR-Cas9 gene essentiality

In CRISPR-Cas9 screening data, each single-guide RNA (sgRNA) in one gene has its unique expression fold change (before knockout versus after knockout), indicating its importance to cell survival. Each gene might have multiple sgRNAs. Since we were looking for drug targets on the gene level, we converted sgRNA level fold-change to gene level fold-change so that each gene will be directly linked to cell survival. The conversion scheme can be described as: For genes that are targeted by only one guide-RNA, we simply used its fold change value as fold change for this gene. For genes that are targeted by multiple sgRNA, we took the average of fold changes of these sgRNAs to represent the overall expression fold change of each gene. We defined gene level expression fold change as ‘gene essentiality value’ in this study. For all 22 pancreatic cell-lines, we calculated the average gene essentiality values among all cell-lines to represent the average gene essentiality values.

## Results

Overlapping 15,664 common genes among 263 gene expression profiles for tumor tissue, normal tissue and cell-line are included for SCNrank analysis, among which 7376 genes are significantly dysregulated by non-paired *t*-test with a *p*-value less than 0.05. 4584 genes out of 7376 genes are significantly over-expressed in the tumor tissues group compared to the normal tissue group. We then mapped the 4584 genes onto the STRING human PPI network. Four thousand one hundred forty-four genes have overlapped with the PPI network. 367 out of 4144 are drug targets of FDA approved drugs. In total, 4141 genes and associated 931,288 pairs of gene-gene interaction of network with 367 FDA approved drugs’ targets (which includes 90 cancer drug targets) are inputs into the SCNrank algorithm to seek potential targets for PDAC patients.

### Potential target subnetworks and targets ranked by SCNrank

In target ranking process, we selected the top 40 eigenvalues for further K-means clustering, which led to 198 clusters (subnetworks) for PDAC patients. One hundred ninety-eight complete clusters and their members can be found in Additional file 1. All 367 targets are scored and ranked by SCNrank system. Table 3 shows the top ten targets and two well-known PDAC drug targets ranked by ‘SCNrank’, of which POLE2 and DHFR are known cancer drug targets. ERBB2 and MTOR are PDAC drug targets. A complete ranked list can be found in Additional file 1.

**Table 3:** Statistics of top-ranked drug targets. Column 2: Ranks by SCNrank Column. 3: cancer drug target information. Column 4: average expression values in tumor tissue samples. Column 5: average expression values in normal tissue samples. Column 6: log2 fold change of expression differences between tumor group and tissue group. Column 7: T value from T-test between tumor and normal group. Column 8: *P*-value from T-test between tumor and normal group. Column 9: gene essentiality value (cell survival rate at T3 versus at T0). Positive values and negative values indicate an enhanced and reduced cell survival rate respectively in vitro

Name	RANK	Cancer drug target (Y/N)	Tumor gene expression (Log2 average)	Normal gene expression (Log2 average)	T_v_N Log2 FC	*T*-value	*p*-value	Gene essentiality in CRISPR
PGK1	1	N	10.18	9.28	0.90	8.03	< 0.01	−1.84
POLE2	1	Y	5.87	4.83	1.04	5.31	< 0.01	−1.31
HMMR	2	N	6.83	5.06	1.77	4.31	< 0.01	−0.96
VDAC1	4	N	9.53	8.83	0.70	6.30	< 0.01	−1.85
PPP2CA	5	N	8.61	8.40	0.21	3.98	< 0.01	−1.94
DARS2	6	N	5.63	5.16	0.47	3.02	< 0.01	−0.54
TK1	7	N	6.56	5.95	0.61	3.37	< 0.01	−0.42
VARS	8	N	5.52	5.14	0.38	3.01	< 0.01	−2.13
DHFR	9	Y	7.09	6.45	0.64	3.75	< 0.01	−1.06
MMP14	10	N	7.40	6.51	0.89	4.37	< 0.01	−0.21
ERBB2	13	Y	6.65	5.58	1.07	3.23	0.01	−0.20
MTOR	32	Y	5.45	5.04	0.44	3.74	< 0.01	−1.24

**Fig. 3:**
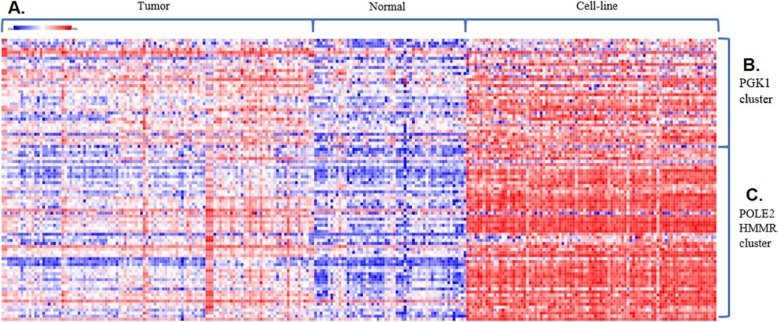
Heatmap of PGK1 and POLE2-HMMR clusters in three different expression profiles. Cluster 1 and 2 refer to PGK1 cluster and POLE2-HMMR cluster respectively. Tumor, Normal and Cell-line indicate tumor samples, normal samples and cell-line samples respectively. Red and Blue color in the panel label indicate over-expression and under-expression of genes respectively

The 12 selected genes are all highly expressed in tumor tissue compared to normal tissue. Moreover, the loss of all 12 genes cause reduced cell survival. Among them, two widely accepted targets ERBB2 and MTOR in treating PDAC are caught by SCNrank algorithm. PGK1, POLE2 and HMMR are the top three ranked targets. PGK1 is in a cluster of 41 genes. POLE2 and HMMR are together in a cluster of 67 genes. Figure 3 shows the expression level of two clusters containing the top three ranked targets in tumor tissue, normal tissue and cell lines. It can be observed that these genes show a concordant high expression pattern in cell-line and tumor groups than in the normal group.

**Fig. 4:**
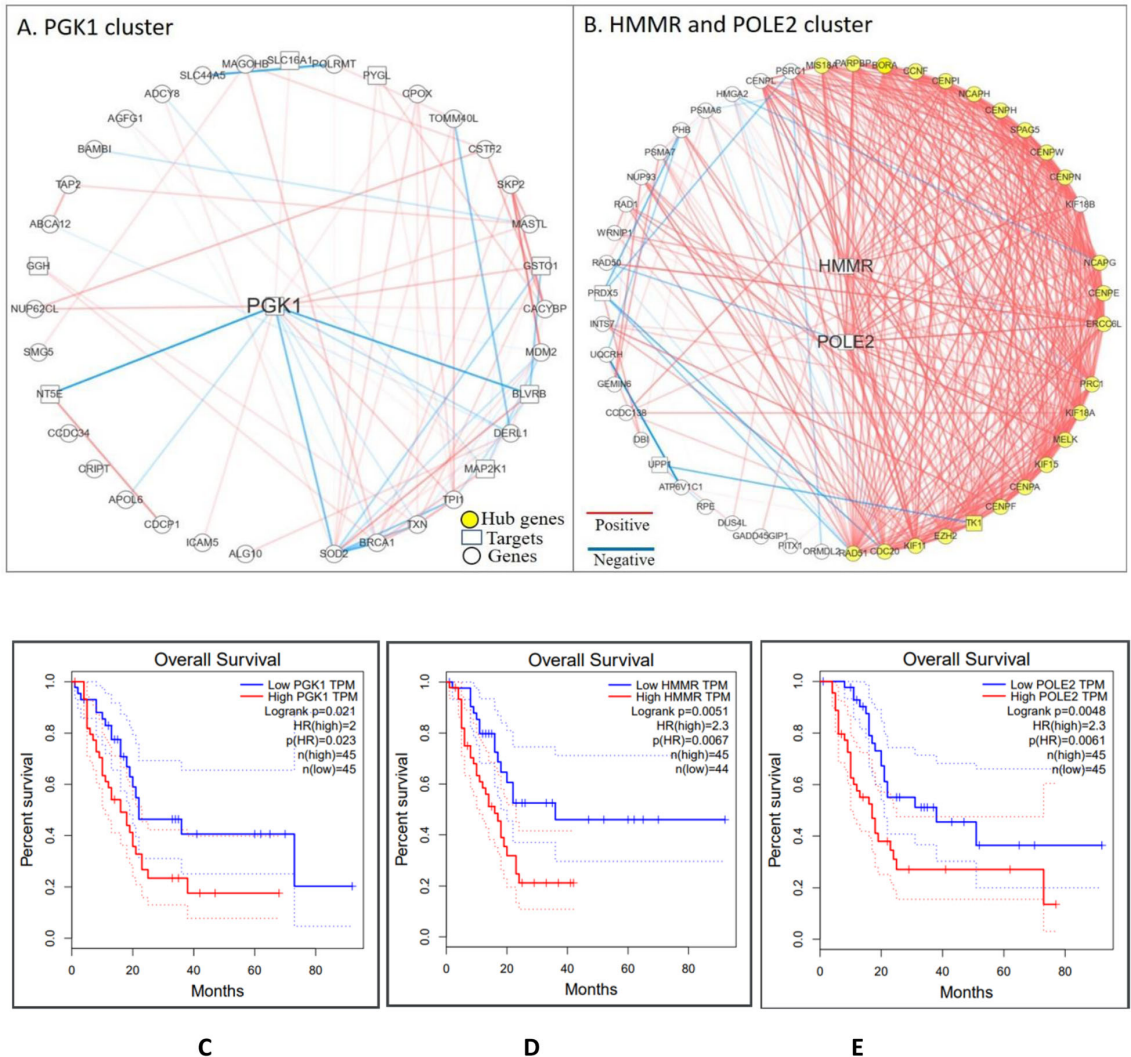
Top three ranked drug targets with their interactions with other nodes in corresponding clusters in cell-line integrated network and the survival analysis on them. In (**a**) and (**b**), cube nodes indicate known targets while the circle nodes indicate other genes. Red and blue lines indicate positive and negative correlations respectively. Line shade indicates correlation intensity. Nodes are placed in a clockwise order by their degrees. **a** Top rated Drug targets ‘PGK1’ and the subnetwork of its cluster. PGK1 is the node that has the highest number of connections. **b** Second and third rated Drug targets ‘POLE2’, ‘HMMR’ and the corresponding subnetwork of their common cluster. Yellow highlighted genes are common genes between HMMR and POLE2. RAD51 is the node that has the highest number of connections. **c** High expression of PGK1 versus Low expression of PGK1 survival curves. **d** High expression of HMMR versus Low expression of HMMR survival curves. **e** High expression of POLE2 versus Low expression of POLE2 survival curves

Glycolytic enzyme phosphoglycerate kinase 1 (PGK1) is a gene that codes for a glycolytic enzyme that catalyzes the synthesis of 3-phosphoglycerate. Its functions and mechanisms are not yet completely understood. As an inhibitor, PGK1 inhibits the secretion of vascular endothelial growth factor (VEGF) and interleukin-8, thus inhibiting Angiogenesis [37]. However, multiple studies have suggested that in metastatic tumor cells, PGK1 plays a completely contrary role. Overexpression of PGK1 facilitates not only tumor growth and interaction with microenvironment, but tumor invasion and metastasis in liver, gastric and prostate cancer [38, 39]. In this study, PGK1 has been identified as the target that can cause the highest influence towards its cluster (shown in Fig. 4a). It interacts not only with the greatest number of genes, but also with the greatest number of other targets in the cluster. Most of its correlations with its neighbors are positive.

DNA Polymerase Epsilon 2, Accessory Subunit (POLE2) is highly involved in DNA repair and replication. It has been previously reported to have a high association with colorectal cancer [40]. In this study, POLE2 is ranked as the second highest target. Even though its cluster is much larger than the cluster of PGK1 (shown in Fig. 4b), the influence of POLE2 towards the whole cluster is not as strong as the influence of PGK1.

Hyaluronan Mediated Motility Receptor (HMMR), which is the target with the third highest score, is highly involved in cell motility. HMMR forms a complex with BRCA1 and BRCA2, thus it has been identified as a high-risk factor in multiple cancer types such as breast cancer and fibrosarcoma [41, 42]. Interestingly, HMMR is in the same cluster with POLE2 (shown in Fig. 4b). Their degrees and ranks are very similar, implying their equal influence towards the whole cluster.

### Pathway enrichment analysis for the top three ranked targets and their clusters

For all 198 clusters, we performed pathway enrichment analysis with ‘Gene Set Enrichment Analysis’ GSEA [43]. We selected ‘C5 go gene sets BP GO biological process’ database version 6.2, which contains 4436 gene sets annotated by GO term with their functions, as a reference and performed functional analysis for each cluster with significance level *P* < 0.05. GSEA analysis required a ranked gene list to perform such analysis, so we used log fold change of tumor vs normal tissue as their weights and ranked them. Complete enriched pathway results and related gene lists can be found in Additional file 1.

Our top-ranked gene, PGK1 with its cluster, has significantly enriched ‘CARBOHYDRATE_CATABOLIC_PROCESS’. The second and third gene, HMMR and POLE2, with their clusters, have significantly enriched multiple pathways such as ‘CELL CYCLE’ and ‘MITOSIS’. These pathways are all highly related to cell cycle and cell division, suggesting these two genes along with their cluster members, are critical components in regulating cell cycles. Moreover, HMMR and POLE2 enriched 8 pathways of 11 total enriched pathways that are enriched by the entire cluster, suggesting common functional activities.

### Ranked targets validation by clinical outcomes

We performed survival analysis for differentially expressed PGK1, HMMR and POLE2 from public database ‘GEPIA’ (http://gepia.cancer-pku.cn/). GEPIA [44] is a public database containing 9736 tumors and 8857 normal samples from TCGA [45] and GTEx [46] projects. In Fig. 4c, d, e), all three targets showed a significant difference (Hazard ratio *P*-value< 0.01) in patients’ survival. Low expression of these three genes provides significantly higher survival than high expression. Survival curves of all three genes show a similar pattern at around 20 months, at which low expression curves start to have clear segregation from high expression curves.

### Targets accordance comparison between clinical drug treatment in pancreatic cancer and selection by SCNrank algorithm

Amanam and Chung systematically investigated all currently available targeted therapies and drug targets for pancreatic cancer [47]. Many studies have reported HER2 overexpression in up to 45% of patients with PDAC [48]. This is due to the fact that HER2 amplifications often occur in PDAC [49]. We mapped the known drug targets to our ranks system and listed result in Table 4.

**Table 4:** Currently available drugs and drug targets for pancreatic cancer comparing associated target ranks from SCNrank algorithm

Targets	Drug	Targets and their ranks
tyrosine kinase		
EGFR	Erlotinib+Gemcitabine	NA
HER2	Trastuzumab	ERBB2 (14)
MAPK	trametinib	MAP2K1 (233)
MTOR	everolimus	MTOR (32)
IGF-IR	Ganitumab	IGF2R (87)
JAK	Ruxolitinib	NA
Angiogenesis		
VEGF	Bevacizumab	PGK1 (1)
Others		
KRAS	Gemcitabine+nab-paclitaxel	NA
DNA repair	Niraparib	NA
Tumor Vaccine	GVAX	NA

In this study, HER2 is ranked 14th by SCNrank. SCNrank covered five commonly used targets in the clinical setting, of which ERBB2 and MTOR are highly ranked (rank 14 and rank 32 respectively). All the missing targets are not included in 4414 genes for constructing integrated networks at the start.

## Discussion

### Research in pancreatic cancer target selection

Recently, drug target selection has been extensively studied and various methods have been developed. For instance, the ‘Connectivity map’ project (C-map) curated expression profiles of human cells exposed to thousands of drugs, which can be served for drug repositioning [17]. Ma et al. developed an algorithm named ‘Met-express’ that combines a gene co-expression network with the human metabolic network to predict drug targets for pancreatic cancer. However, these methods only utilize expression data as fundamental knowledge and incorporate other biological knowledge to predict targets. However, most drugs function on protein level eventually. And expression level regulation might not eventually reflect on protein level. Secondly, their analysis lacks the support of cell survival phenotypes that directly reflect the effects of gene knockdown/knockout experiment. To our knowledge, SCNrank is the first algorithm that can incorporate expression data, PPI data and gene perturbation data (CRISPR or RNAi) for selecting and ranking drug targets. The novelty of the SCNrank algorithm mainly reflects in: i. SCNrank is the first algorithm that takes advantage of dimension reduction methods to integrate three different types of omics data into a comprehensive network for drug target selection; ii. SCNrank ultilized CRISPR data to benefit the target selection. The CRISPR data can mimic the real drug response of drugs; iii. SCNrank uses spectral clustering to reduce data dimensions to capture features on tissue-based omics-data and ranks drug targets on cell-line omics-data, which makes the target selection process more reliable. Spectral clustering was initially introduced to cancer biology for identifying novel subtypes of Triple Negative Breast Cancer (TNBC) [50]. To our knowledge, it has never been used for selecting genotypic features from an integrated network. Despite the advantages, there is still room for SCNrank to improve. The possible future might include i. incorporate pathway information into target selection process for PDAC. Pathway information provides a different perspective in understanding the progression and treatment of PDAC [45, 51, 52]. Targeting cancer related pathways can be a highly effective strategy for treating PDAC. Thus, it is necessary to incorporate pathway information into the drug target ranking and selection process; ii. Incorporate functional information into the target selection process. SCNrank algorithm ranked drug targets mainly based on differential expression, protein-protein interaction and tissue-target concordance. However, different proteins might have different docking capacities, which directly affects their potential to become a druggable target. Unfortunately, SCNrank algorithm doesn’t take this information into account for ranking targets. Integrating this information into the whole process is necessary.

### Clinical targets of drug in pancreatic cancer

Tumor cells prefer glycolysis to oxidative phosphorylation for providing energy during proliferation and metastasis. This phenomenon is called the ‘Warburg Effect’ [53] and often occurs in certain tumor types such as brain cancer, liver cancer and pancreatic cancer. PGK1 is an important enzyme in the metabolic pathways. Recent studies have revealed that PGK1 can promote cell proliferation and tumorigenesis by enhancing the Warburg effect. For instance, Li et al.’s study reveals that PGK1 functions as a protein kinase to phosphorylate PDHK1, which further promotes the Warburg effect in brain tumorigenesis [54]. Hu et al. recently reported that acetylation of PGK1 can promote cell proliferation and tumorigenesis in liver cancer via glycolysis pathways [55]. Xie et al.’s study has pointed out that PGK1 is highly involved in MYC-induced metabolic reprogramming, which further causes a reinforced Warburg effect [56]. From the pathway analysis result from section 3.3, we also observed a significantly enriched ‘cellular metabolic process’ pathway, which implies the activated Warburg effect in our PDAC samples. So far, there are studies that focus on targeting the Warburg effect to treat pancreatic cancers. Rajeshkumar et al. has selected a small molecule called ‘FX11’, which inhibits a lactate dehydrogenase-A (LDH-A), a critical enzyme in metabolizing pyruvate, to block the Warburg effect [57]. They observed that for TP53 mutant cells, their approach can significantly increase tumor cell apoptosis. These studies provide the possibilities of targeting the Warburg effect to treat PDAC. Hence, together with the survival analysis result shown in Fig. 4a, our findings suggested that PGK1 is a potential target that alternatively targets the ‘Warburg Effects’ and thus is worth further experimental validation.

‘DNA polymerase epsilon 2’ (POLE2) and ‘Hyaluronan-mediated motility receptor’ (HMMR) have been previously reported as significantly hyper-expressed in both PDAC tissues and cell-line expression profiles [58]. Studies have linked HMMR and its product ‘Receptor for Hyaluronan Mediated Motility’ (RHAMM) to a variety of hematological malignancies and other solid tumors [59–61]. This is because RHAMM, working in concert with BRCA1 and BRAC2, can significantly promote tumor growth and metastasis for pancreatic cancer [62] in vivo, and multiple other cancer types such as basal-like breast cancer [63] and glioma [64] in vivo. Hence, Willemen et al. pointed out of HMMR/RHAMM being a considerable potential target for cancer immunotherapy [65]. Moreover, Li, Ji and Wang have targeted HMMR via long noncoding RNA (lncRNA) and successfully suppressed Glioblastoma in mouse xenograft model [66]. This evidence suggests that HMMR and its product RHAMM is worth further study in its potential to be used as a PDAC drug target. POLE2 is highly involved in DNA repair and replication. However, targeting POLE2 to treat cancer is rarely reported. Li et al. used β-elemene, which is a type of elemane sesquiterpenoids, to suppress POLE2 expression and restrain lung adenocarcinoma cell malignant in vitro [67], which could be used as evidence of treating pancreatic adenocarcinoma (PDAC) by targeting POLE2.

## Conclusion

In this study, we developed an algorithm called ‘SCNrank’ that links cell lines CRISPR technology with gene expression profiles and the PPI network to score and rank drug targets for PDAC. We utilized cutting edge dimension reduction methods and network analysis methods to identify the potential targets. We disclosed the molecular mechanism of potential disease genes in PDAC and roles systematically by performing pathway enrichment analysis. We validated our top-ranked genes by comparing them with existing pancreatic cancer drug targets and performing survival analysis on top-ranked targets to predict their clinical outcomes. We showed that the top-ranked target, PGK1, plays a key role in tumor cell glycolysis in PDAC and has high potential as a target for treating PDAC. Our second and third-ranked targets, POLE2 and HMMR have been proven to promote PDAC and various other cancer types. Moreover, HMMR has been extensively studied as a target for treating lung adenocarcinoma and glioma. This might serve as evidence of using HMMR as a novel drug target for PDAC. Taken together, the results provide new guidance for future clinical treatments.

## Supplementary information

**Additional file 1.** Information of all samples, identified clusters and ranked targets: S1: sample annotation; S2: complete ranked target list; S3: identified clusters and their members; S4: targets with clusters.
